# Supplementary material for: Analysis of CFTR mRNA and Protein in Peripheral Blood Mononuclear Cells via Quantitative Real-Time PCR and Western Blot
Source: Int J Mol Sci. 2024 Jun 8;25(12):6367. doi: 10.3390/ijms25126367 (PMC11203434; doi:10.3390/ijms25126367)
Supplement: Supplementary file 1 [file ijms-25-06367-s001.zip › ijms-2811747-supplementary.pdf]

Supplementary Appendix to Schnell et al. “Analysis of CFTR mRNA and protein in immune cells via quantitative real-time PCR and Western blot.”

This supplementary Appendix is provided by the authors to support the work by Schnell et al. “Analysis of CFTR mRNA and protein in immune cells via quantitative real-time PCR and Western blot.”

Supplementary Appendix to Schnell et al. “Analysis of CFTR mRNA and protein in immune cells via quantitative real-time PCR and Western blot.”

Table of contents

Supplementary Figures 3

Supplementary Figure S1 3

Supplementary Figure S2 4

Supplementary Figure S3 5

Supplementary Figure S4 6

Supplementary Figure S5 7

Supplementary Figure S6 8

Supplementary Figure S7 9

Supplementary Figure S8 10

Supplementary Figures

Supplementary Figure S1

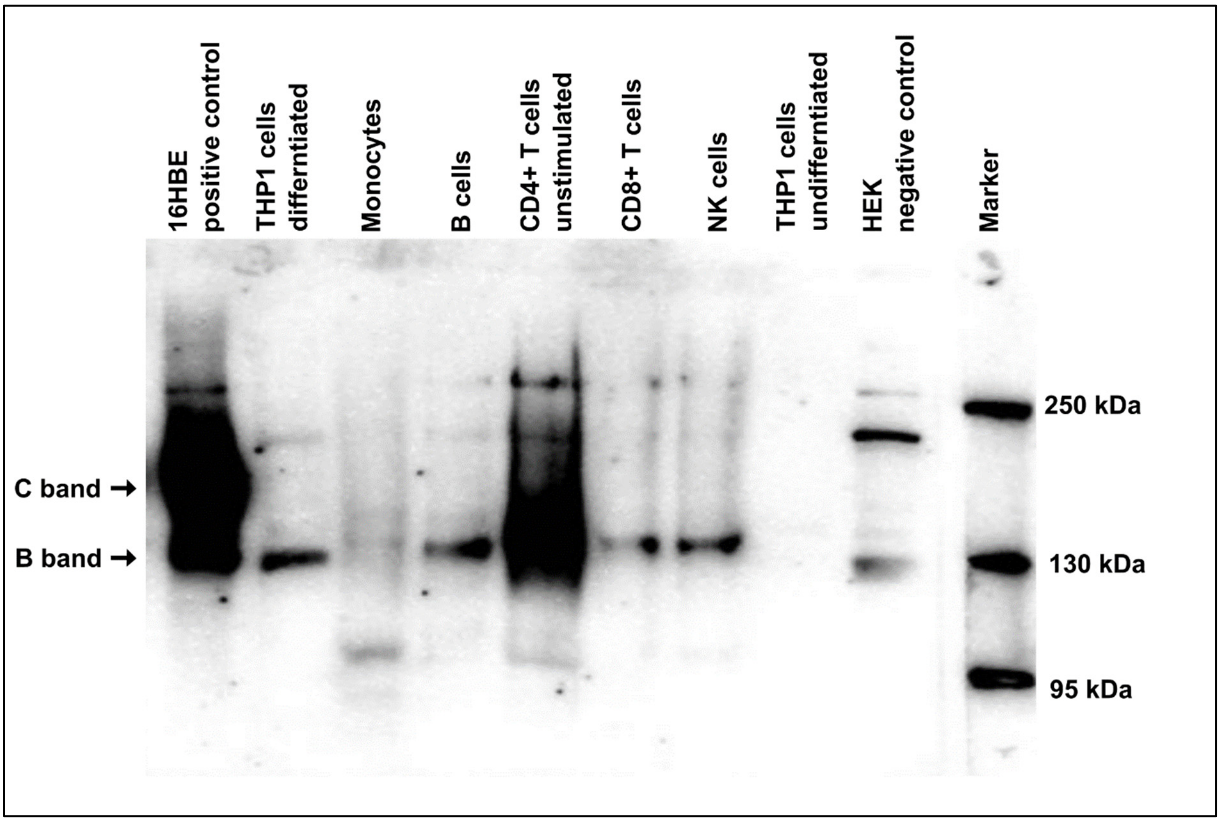

Supplementary Figure S1: Source data for Figure 1. Primary immune cells (monocytes, NK cells, B cells, CD4+ and CD8 + T-cells) isolated of the peripheral blood of a healthy subject were compared regarding their CFTR expression with undifferentiated, monocyte-like THP1 cells and differentiated, macrophage-like THP1 cells. In Figure 1, undifferentiated THP1 cells are not shown, as we detected no signal at all in the CFTR or Vinculin blots, probably due to a faulty membrane loading.

Supplementary Appendix to Schnell et al. “Analysis of CFTR mRNA and protein in immune cells via quantitative real-time PCR and Western blot.”

Supplementary Figure S2

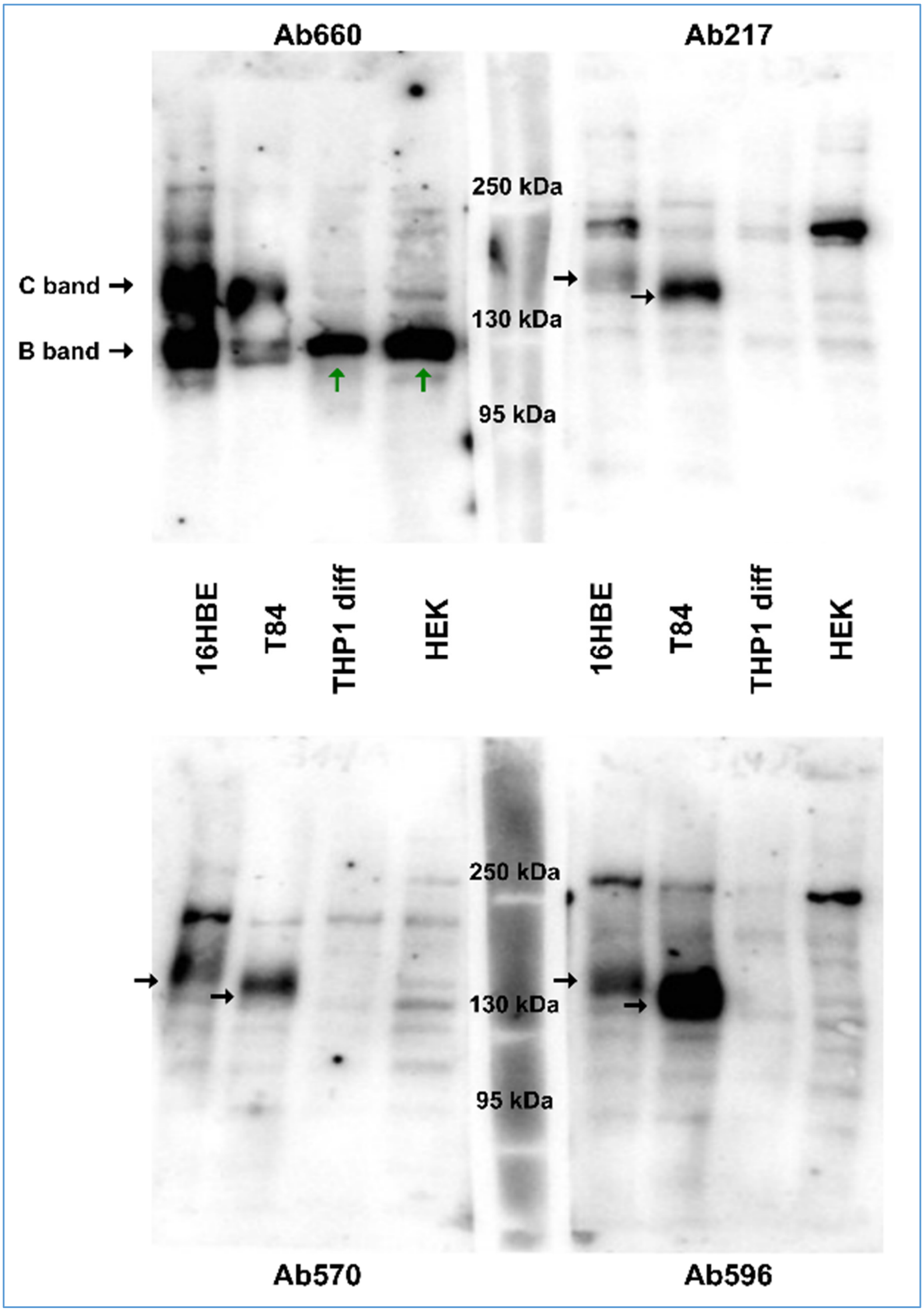

Supplementary Figure S2: Separate immunoblots for each CFTR antibody (Ab660, Ab217, Ab570 and Ab596) show the distinctive CFTR-specific C-band in CFTR expressing cell lysates (black arrows in 16HBE14o—cells, T84 cells) whereas the Ab660 raises a strong signal at 130kDA in differentiated THP1 and CFTR-negative HEK cell lysates (green arrow).

Supplementary Figure S3

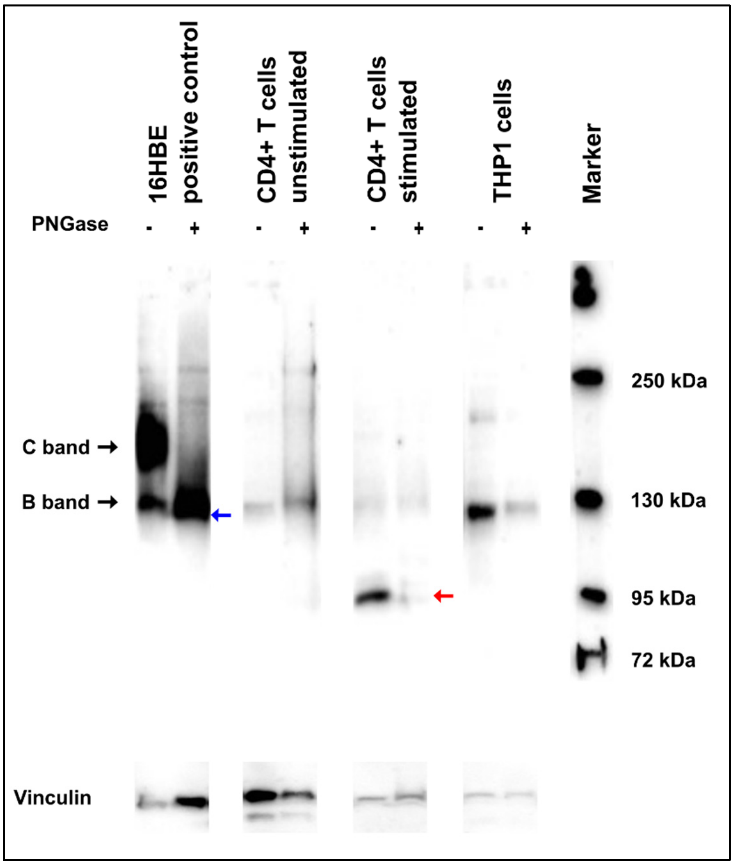

Supplementary Figure S3: Glycolytic PNGase digest in immune cells: An immunoreactive band similar in size to CFTR-B band could be observed in differentiated THP1 cells, stimulated and unstimulated CD4$^+$ Tcells and HEK cells at 130kDa (green arrow). After PNGase digest, 16HBE14o- showed a typical shift of CFTR-B and -C bands towards the CFTR-A (blue arrow) whereas the band signal remained unchanged in the other cell types. At smaller and fainter band at 95 kDa observed in stimulated CD4$^+$ T cells (red arrow) resolved after PNGase digest. Vinculin was used as a loading control.

Supplementary Appendix to Schnell et al. “Analysis of CFTR mRNA and protein in immune cells via quantitative real-time PCR and Western blot.”

Supplementary Figure S4

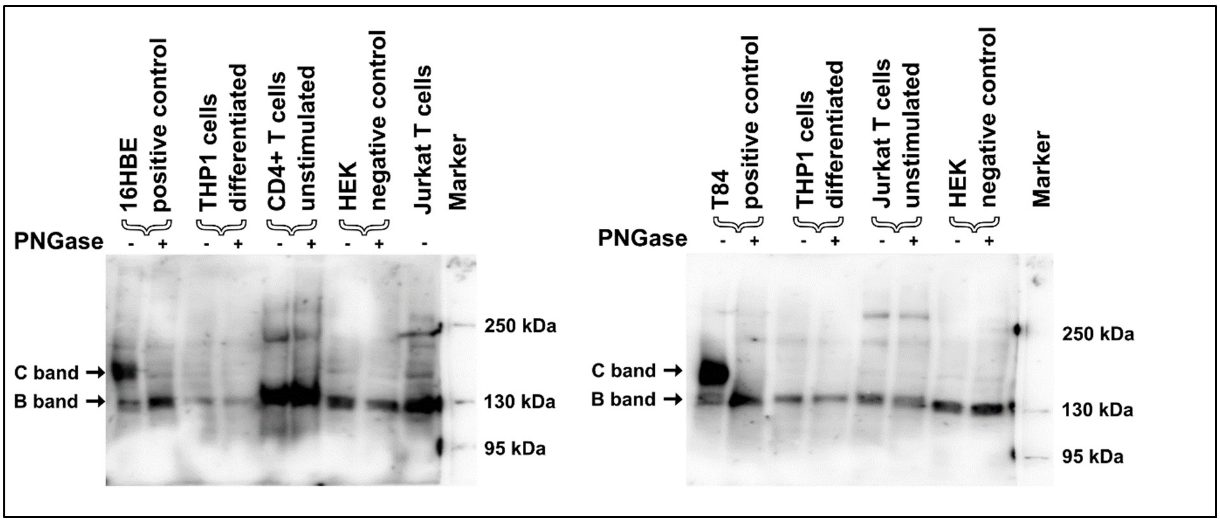

Supplementary Figure S4: Source data for Figure 2.

Supplementary Figure S5

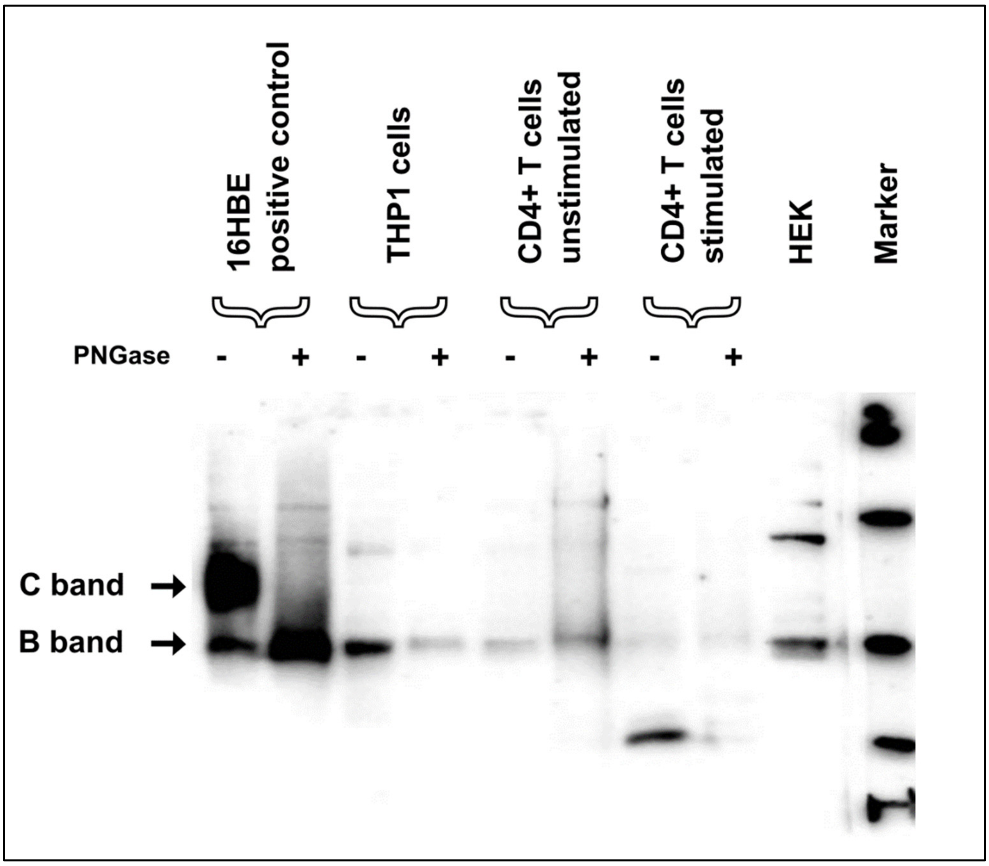

Supplementary Figure S5: Source data for Figure 2. 16HBE14o-- cells, differentiated THP1 cells as simulated and unstimulated CD4+ are shown with or without glycolytic PNGase digest. HEK cells are not shown in Figure 2 as they served only as control lane and were not digested.

Supplementary Appendix to Schnell et al. "Analysis of CFTR mRNA and protein in immune cells via quantitative real-time PCR and Western blot."

Supplementary Figure S6

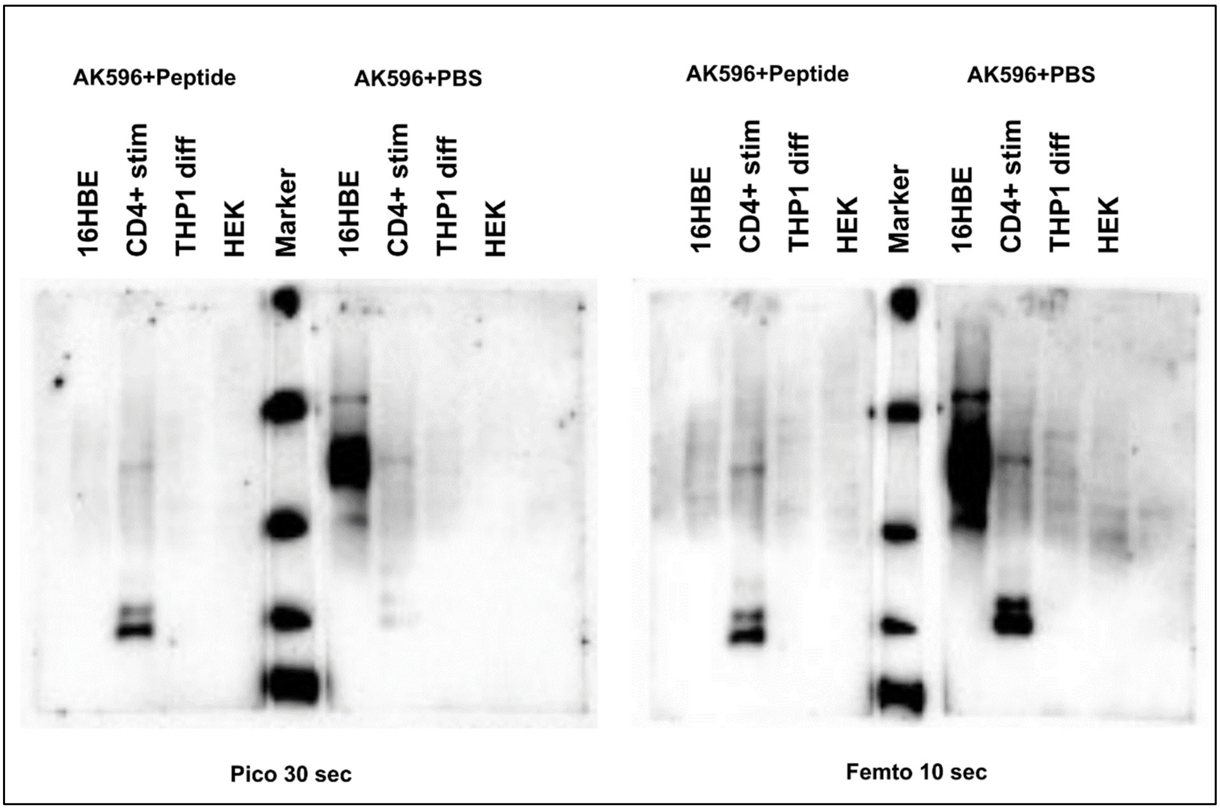

Supplementary Figure S6: Source data for Figure 3. The figure shown in the manuscript as figure 3 is derived from a single gel and a single western-blot membrane prepared thereof. While the signals for vinculin can be derived from a single exposure, the CFTR amounts may vary considerably between sources. Therefore, to detect CFTR from 16HBE14o-, 30 sec exposure to SuperSignal West Pico was sufficient, while most immunoreactive bands from immune cells can only be seen with SuperSignal West Femto at 10 seconds or longer exposure times.

Supplementary Figure S7

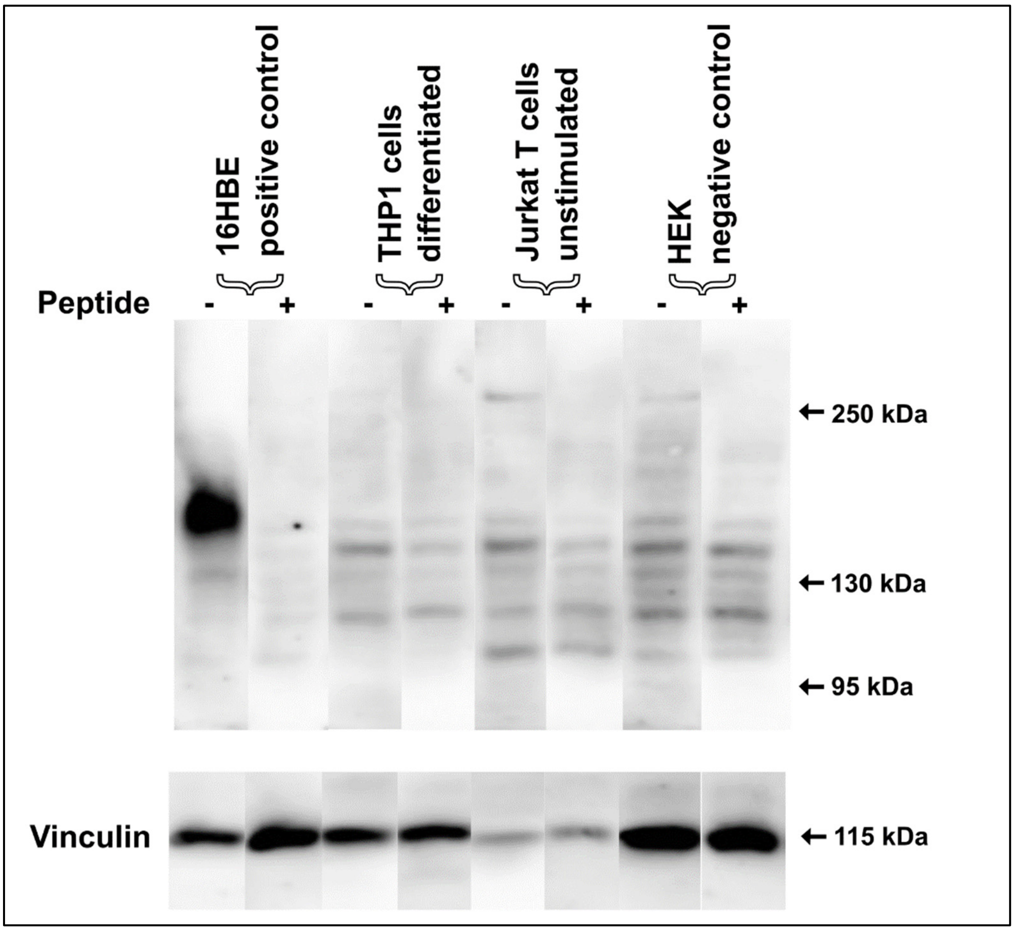

Supplementary Figure S7: Peptide competition of the CFTR-specific antibody Ab 596 in Jurkat T cells. In contrast to 16HBE14o- cells - irrespective of whether or not competing peptide is used to preincubate the antibody directed against CFTR - band patterns are identical for Jurkat T cells, THP1 cells and HEK cells, demonstrating that those immunoreactive signals are not generated by the antibody binding to CFTR protein.

Supplementary Appendix to Schnell et al. “Analysis of CFTR mRNA and protein in immune cells via quantitative real-time PCR and Western blot.”

Supplementary Figure S8

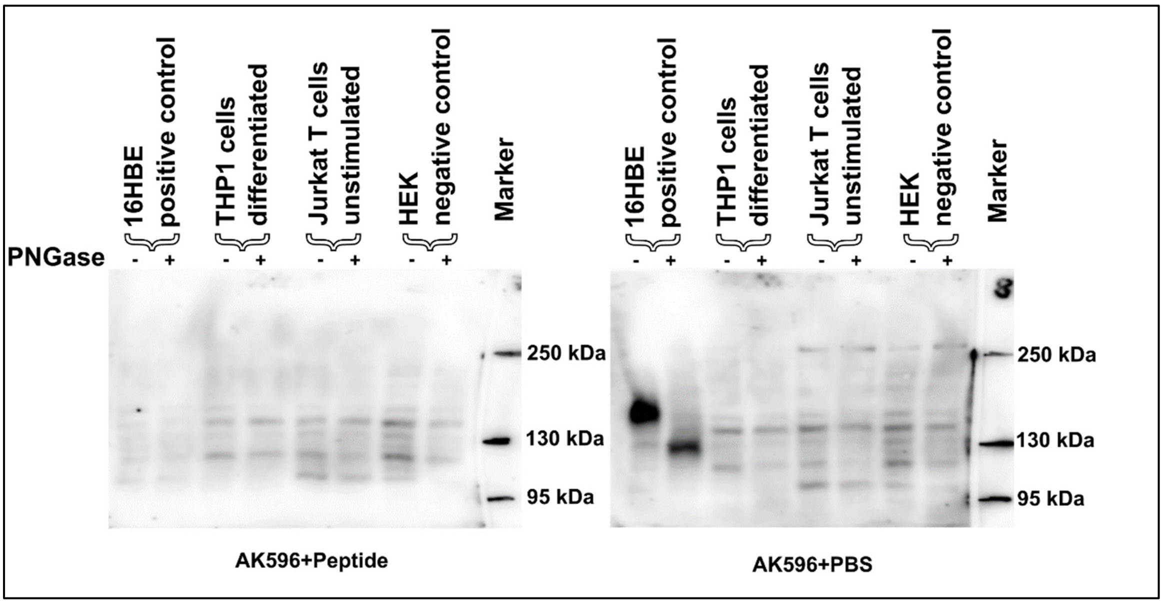

Supplementary Figure S8: Source data for Supplementary Figure 6.
